# In silico design of MHC class I high binding affinity peptides through motifs activation map

**DOI:** 10.1186/s12859-018-2517-3

**Published:** 2018-12-31

**Authors:** Zhoujian Xiao, Yuwei Zhang, Runsheng Yu, Yin Chen, Xiaosen Jiang, Ziwei Wang, Shuaicheng Li

**Affiliations:** 1 South China Normal University, HEMC (Guangzhou Higher Education Mega Center) campus, GuangZhou, China; 2 Hunan Agricultural University, Nongda Road, ChangSha, China; 3 Huazhong University of Science and Technology, Luoyu Road 1037, Wuhan, China; 4 City University of Hong Kong, 83 Tat Chee Ave, Kowloon Tong, Hong Kong, China

**Keywords:** Design new peptides with high binding affinity to MHC-I molecule, Convolutional neural network, Motifs activation map

## Abstract

**Background:**

Finding peptides with high binding affinity to Class I major histocompatibility complex (MHC-I) attracts intensive research, and it serves a crucial part of developing a better vaccine for precision medicine. Traditional methods cost highly for designing such peptides. The advancement of computational approaches reduces the cost of new drug discovery dramatically. Compared with flourishing computational drug discovery area, the immunology area lacks tools focused on in silico design for the peptides with high binding affinity. Attributed to the ever-expanding amount of MHC-peptides binding data, it enables the tremendous influx of deep learning techniques for modeling MHC-peptides binding. To leverage the availability of these data, it is of great significance to find MHC-peptides binding specificities. The binding motifs are one of the key components to decide the MHC-peptides combination, which generally refer to a combination of some certain amino acids at certain sites which highly contribute to the binding affinity.

**Result:**

In this work, we propose the Motif Activation Mapping (MAM) network for MHC-I and peptides binding to extract motifs from peptides. Then, we substitute amino acid randomly according to the motifs for generating peptides with high affinity. We demonstrated the MAM network could extract motifs which are the features of peptides of highly binding affinities, as well as generate peptides with high-affinities; that is, 0.859 for HLA-A*0201, 0.75 for HLA-A*0206, 0.92 for HLA-B*2702, 0.9 for HLA-A*6802 and 0.839 for Mamu-A1*001:01. Besides, its binding prediction result reaches the state of the art. The experiment also reveals the network is appropriate for most MHC-I with transfer learning.

**Conclusions:**

We design the MAM network to extract the motifs from MHC-peptides binding through prediction, which are proved to generate the peptides with high binding affinity successfully. The new peptides preserve the motifs but vary in sequences.

## Background

### Introduction

The genetic heterogeneities and polymorphisms across different individuals contribute substantial factors of different responses to the same drug or medicine. One of the ultimate goals of the precision medicine is hence to fabricate personized medicines. The human major histocompatibility complex (MHC), coded by a region on chromosome six, serves essential roles in the immune system and this region is highly polymorphic. The MHC gene family code a class of proteins, which are often referred to as MHC molecules. They recognize and bind to antigenic peptides (the binding moiety is called epitope) and present it to the cell surface for interacting with TCR (T cells receptor), then induce the immune response [1]. MHC gene family consists of three subgroups, class I, class II, and class III. MHC-I and MHC-II bind with specific peptides. MHC-I molecules have closed ends so that the specific binding peptide fragments only contain 8-11 residues. MHC-II molecules have open ends and bind longer peptide fragments, which usually contains 14-18 residues. MHC-II-peptides binding is more complicated to model due to the groove of MHC-II only matches a portion of the peptide called binding core.

Studying the specific features of MHC-peptides binding is of great significance to understand the mechanisms of immune response, develop immune epitopes and drug discovery [2]. Due to the high cost and complicate preprocessing in the experimental method, in recent years, various machine learning algorithms are widely applied to extract binding features. Meanwhile, increased computational power and data availability boost the adhibition of deep learning. Deep learning is developing rapidly and now is with increasing importance in the field of biomedicine [3]. For example, in proteomics field, Pcons2 [4] and Deep-RBPPred [5] are proposed; in predicting enhancers and regulatory regions, DanQ [6], Basset [7], DeepSEA [8] and DeepMotif [9] *etc* are proposed. Notably, researchers prefer deep learning to predict the binding affinity between the peptide and MHC and proposed different neural networks such as HLA-CNN [10], MHC nuggets [11], MHCflurry [12] and netMHCpan [13] in recent years.

Another important perspective is binding motifs. These motifs are characterized primarily by the requirement for a few properly spaced and essential primary anchor residues [14].

Here, we propose an MHC and peptide binding Motif Activation Mapping Network (MAM Network) to **generate new peptides of high binding affinity in silico** with the binding prediction and binding activation map. In the binding prediction and activation map, we predict whether the peptide is a binder (or non-binder) and calculate the contribution of each site to the binding affinities. To generate peptides, we substitute amino acid at the position with the lowest score according to the contributions.

Our model incorporates several important features. We emphasis fine-tune application in transfer learning when extended to another which can be extended to multiple types of MHC. Further, generating new high-affinity peptides cannot only expand the present data set but also provide large resources for further studies.

In summary, we propose here:

1. A well-performed binding probability prediction network called MHC-CNN which reaches to state of the art.
2. A novel motifs activation map model that build the mapping from components of the peptide to its binding probability with MHC molecule.
3. Two transfer learning methods were applying on prediction and generation of small datasets, which also reveals the similarities of binding mechanism among various MHC molecules.
4. A well-performed generator that can generate brand-new peptides with high affinity.

### Related work

Researchers study the MHC-peptides interaction for decades, the obtained insights advance in our understanding of the immune system, scientific treatment of diseases and the development of new drugs.

#### Binding affinity prediction

Existing related works are mainly on binding affinity prediction. Reach et al. (2002) [15] propose PSSM (Position-specific scoring matrix) for predicting the MHC-peptides binding affinity and conducted a preliminary test. The PSSM is a representative matrix, which is the cornerstone of MHC-peptides binding research. Based on MHC class II has more complicated binding pattern than class I, the peptides are longer and more difficult to predict. Nielsen et al. (2004) [16] propose the Gibbs sampling method for the prediction of MHC-II-peptides binding affinities. Peters and Sette (2005) [17] supplement the SMM algorithm (Stabilized Matrix Method) and transform the binding affinity prediction problem into a matrix-vector regression problem.

Hidden Markov Models (HMM), Support Vector Machines (SVM) and artificial neural networks (ANN), are also developed for binding affinities prediction. Machine learning algorithms can build more complex nonlinear models to achieve better prediction performance in the MHC-peptides binding affinity prediction. For example, ANN can capture the complex inter-relationships in the non-linearity in the s, which is suitable for classification and recognition tasks as well as motifs extraction. ANN-based prediction models have emerged such as netMHCpan [13], netMHCIIpan [18] and MHCflurry [12]. Most of these models only include one or two full-connect layers, with the optimizing different network structures and parameters, ANNs take advantages of flexibility and adaptability. ANN approaches are outstanding for its accuracy, but lack of explanatory. In the field of MHC binding, the HLA-CNN [10] which uses three convolutional layers and two fully-connected layers with word embedding for encoding, leading to the total accuracy is over all the traditional methods and shallow neural networks. MHCpred [19] with the structure of deep char-RNN, which applies three LSTM (Long Short-Term Memory) layers and adaptively finding the appropriate parameters to enable the model to learn the hidden features more efficiently. The convMHC [20], which uses more than three convolutional layers and inputs MHC sequence and its 3D structure data as supplementary information to predict the binding domain of MHC molecule. Similarly, in the broader field of protein-ligand prediction, Matthew R uses deep CNN model to pose prediction and virtual screening by 3D-structure data and chemical data [21].

#### Deep learning in pharmacy design

Biomarker identification and drug design are the emerging fields for deep learning application [22]. Molecular modeling based on deep learning could generate a large number of potential and useful compounds, mainly reducing both cost and time than the traditional methods. Increasing data availability reveals deep learning is a promising way to design new drugs effectively. In published researches, generation of the new drug with deep learning has achieved encouraging results. Such as Dru-GAN, produce compelling medicines in PubChem [23, 24] using autoencoder and molecular fingerprinter information. Marwin et al. attempt to use RNN and Q-learning to generate new molecular [25]. In the field of chemical synthesis, using HMMs to simulate the homology molecular is a general way of creating a molecular [26]. It is also getting essential to use the attention model to search for the essential structure in chemical reaction [27, 28].

As far as we know, the generation of potent peptides has not been studied yet but there are a lot of works researching the specific MHC-peptides binding motifs. NNAlign is a method that has been used for the identification of linear motifs in biological sequences [29]. Deepfit [30] also is used to predict motifs in DNA. Bruno et al. propose a method to predict the motif of the peptide by MS (mass spectrometry) data in MHC-peptide binding field [31].

## Methods

First, we collect the data and filter out the invalid data and noise. To the proposed model for a certain MHC, we divide the peptides belongs to the MHC into training and testing. Then, we represent each amino acid of the peptides with a 15-dimension vector, and thus, represent each peptide of k residues into a 15 ×*k* matrix. With this representation, we training binary classifiers with different random initialization and then average all the trained models. To generate new peptides, we extract weights from the trained network and calculate the contributions to the binding affinities of each amino acid at each site. Then, we generate new peptides according to the mutation methods. Besides, we apply the transfer learning to the well-trained network to other alleles small datasets with fine-tune or zero-shot strategy.

**Fig. 1:**
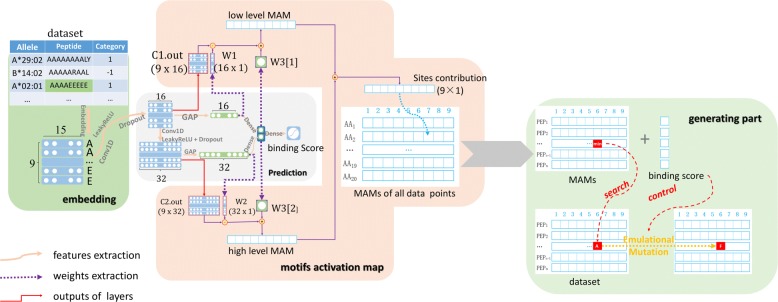
Pipeline of our Motifs Activation Map network. Embedding step is to encode each amino acid into a 15-dimension vector. Prediction step is to predict the binding probability from 0 (non-binder) to 1 (binder) and the weights of MAM network. Our MAM network is to calculate the contribution scores at each site then generate new peptides with mutating the amino acid with lowest contribution score. Here we take 9-mer as an example

We propose the MHC-peptide binding motifs activation mapping network (MAM network) which can learn the weights through the binder vs. non-binder prediction training process then map the weights to extract binding motifs and generate new peptides. As shown in Fig. 1, the framework of our network mainly consists of Embedding, prediction, generation steps. Now we will introduce the details of our network.

### Embedding

The one-hot encoding method or the k-mer encoding method in deep learning have disadvantages that the results are too sparse to converge or too simple to carry characteristics. Therefore, deep learning needs more suitable encoding methods, a recently encoding method in NLP has been used in many fields, such as word embedding [32–35], which is a non-sparse coding method that takes contextual information into account. Word embedding has been proven to be the most efficient encoding method among various encoding methods in deep learning [36]. Consequently, followed by Vang et al. [10], we encode each amino acid into a 15-dimension vector and transfer a set of peptides into a matrix of batch_size ×peptide_length × 15.

### MHC-CNN predictor

**Fig. 2:**
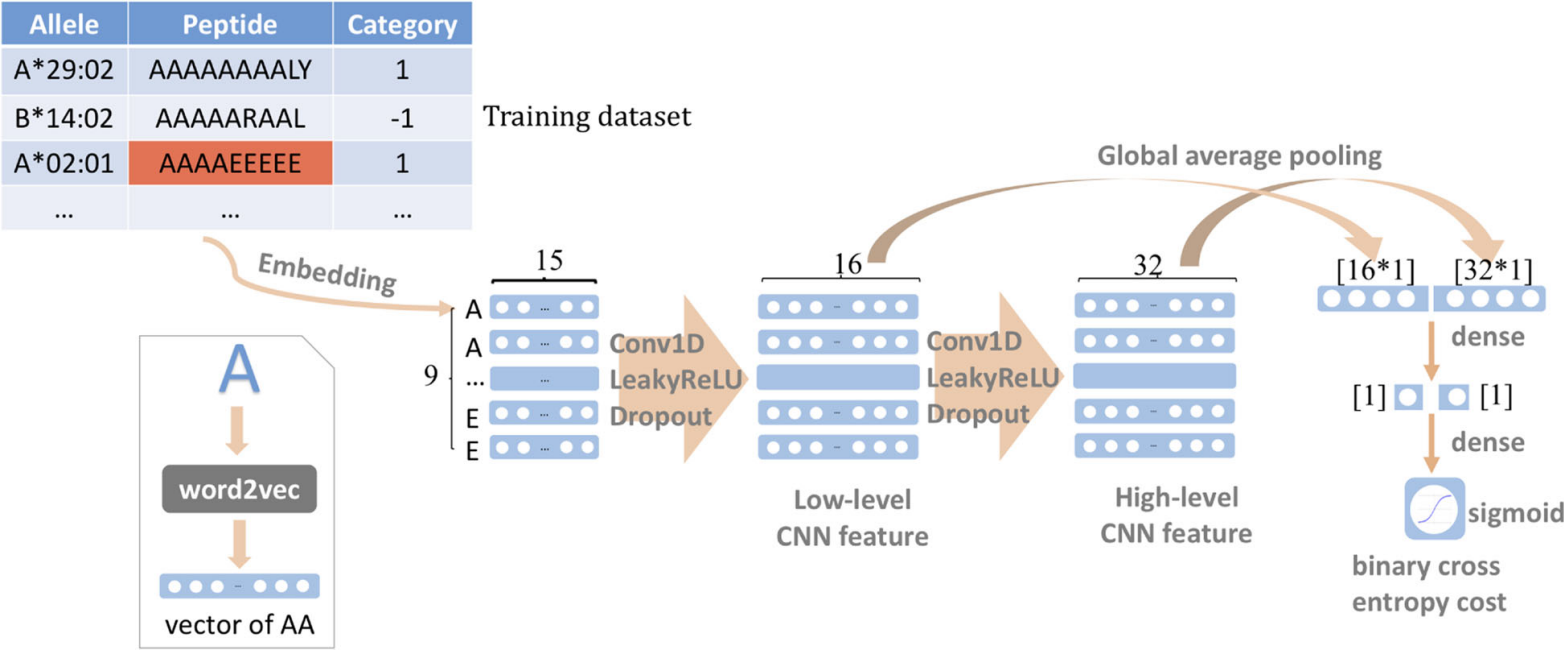
MHC-CNN predictor (The prediction part of our MAM Network). Two 1-D convolution layers are used to extract the hidden features. Global average pooling layer is to replace fully-connected layer and calculate the weights of every feature. Then two dense layer is to merge the features from two levels into one final binding score. The input of our predictor is peptide’s representation matrix while the output is the binding probability. Here we take 9-mer as an example

As shown in Fig. 2, MHC-CNN predictor consists of the following components.

#### Convolutional layer

Binding motifs are critical to the MHC-peptide binding affinities and many methods are proposed to identify the motifs [37–39]. However, the existing sequence-based methods are incapable to recognize and locate these motifs well. One of the main reasons is that these motifs are convoluted and cryptic: sites may have a tight connection with adjacent sites. Therefore, to extract these motifs, we adopt the CNN to analyze the peptide sequences comprehensibly. CNN-based method is adopted to extract feature including the spatial relationship in computer vision [30, 40]. Notably, CNN-based networks are already applied to the prediction of MHC-peptides binding affinity [20, 41]. Nevertheless, existing studies only focus on high accuracy without uncovering the binding mechanisms which the network learned. We ought to focus on the interpretability of the network.

When deciding on the layers of the network, an important issue is to take the overfitting into account. Due to the shortage of data, a deep learning method should be cautiously applied to avoid overfitting. Therefore, we apply a shallow network. The network contains two one-dimension (1-D) convolutional layers representing features from a low level and high level, respectively. The first convolutional layer contains 16 filters and second convolutional layers contain 32 filters. The strides and kernel sizes of both layers are one and seven. These values are small due to that the length of peptides binding to MHC-I is usually short.

#### Global average pooling layer

Many CNN networks adopt the fully-connected (FC) layer after the convolutional layers. However, fully-connected layer mitigates the spatial features [42]. Besides, it is hard to explain the fully connected layers which will lead to the black box problems. To preserve the ability of localization in convolutional layers and meanwhile to avoid the loss of explainability, we decide to use global average pooling (GAP) layer instead of fully connected layer.

A GAP layer has the following advantages. First, we can interpret how each filter contributes to the MHC-peptides binding affinity. Second, it reduces a large number of parameters of the fully connected layer and thus reduces the risk of overfitting. Third, it makes no restrict to the size of input data, which denotes that we can use this work to deal with the peptide with any length while the fully connected layer can only adopt one certain dimension. The GAP network is represented by the formula below:

1 $$  c = \sum\limits_{j} (M_{j} \cdot p(F_{j})),  $$

where *c* denotes the total feature contributions of a certain level while *M*_*j*_ denotes the contribution weights. Function *p*(.) is 1*1 pooling layer and *F*_*j*_ denotes the *j*^*th*^ feature in last convolutional layer. The contribution parameters are learned by backpropagation.

We use a dense layer, which owns one weight without bias as our GAP layer.

#### Multi-level Feature combination

Prior experiments indicate the high-level hidden features solely cannot address the prediction problem and generation process well. It may be due to that the high-level hidden features (or tight features) do not reveal the real motifs completely. Hence, multi-level features need to be applied to our network. To better incorporate the various features’ contribution from different levels, we apply the voting method [43] to merge different level hidden features. The multi-level merging model is given by

2 $$  P = sigmoid\left(\sum\limits_{i} W_{i} \cdot c_{i}\right),  $$

where *P* is the final predicted probability of assuming that this peptide is a binder to the certain MHC molecule. The value of P ranges from 0 to 1; where the peptide is predicted as the binder when the *P* value approaches 1 and as non-binder when the *P* value approaches 0. *W*_*i*_ denotes the weight for *i*^*th*^ level hidden feature while *c*_*i*_ denotes the *i*^*th*^ hidden feature. *sigmoid*(.) stands for the activation function as sigmoid function.

#### Model averaging merge

In this study, we train multiple models with different random initialization and save the graph when there’s no improvement for training. Then we choose the averaging method to merge both prediction results and site scores results.

#### Loss function

Since we aim to extract the motifs through learning, we only use 0 and 1 to represent the peptide is binder (which IC50, an experimental measurement to quantify the binding affinity, is less than 500 nM) or non-binder (which IC50 is more than 500 nM). In this way, the binding probability prediction will be a binary classifier. The binary cross-entropy [44] loss function is chosen for our network loss function.

### Generation

Binding motifs will be essential to generate peptides with high binding affinity. The first step is to calculate the contribution of each amino acid of peptides at each position. We extract the weights from the well-trained network for mapping the contribution vector to the binding affinity, which is shown as the weights extraction flow in Fig. 1 (the dotted line in purple).

#### Motifs activation map layer

**Fig. 3:**
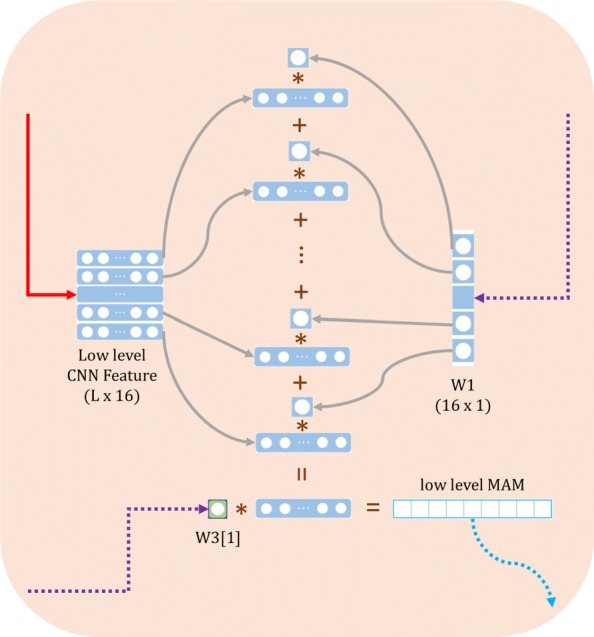
The visualization of our low-level Motifs Activation Map network. Take the example of one 9-mer peptide, converting to the feature matrix with the shape of 9 × 16 (16 is the kernel size of the first 1-D convolutional layer) out from the first convolutional layer. The representation of the site, 9, is preserved. Then using the W1 matrix to add each feature from the low-level weight and collect together. Then the feature matrix of size 9 × 1. As the low-level feature with respect to sites, to get the final site rank, we give a weight for low level and then merge all levels feature matrices together. the final result’s shape is still 9*1, we preserve the length through the calculating of sites contribution vector and it provides intuitive information for us to compare the contribution to the binding probability of each site

When the neural network learns the contribution weights in a different level and different features, how to construct a map (or a connection) from the hidden features to the contributions of each site is important. The high contribution of the site reveals the motifs in the peptide-MHC binding mechanism. Inspired by Class Activation Map method [45], we design Motifs Activation Map layer(MAM layer) according to the formula below:

3 $$  S_{k} = \sum\limits_{i} m_{i} \cdot \sum\limits_{j} (W_{ij} \cdot (F_{ijk}))  $$

4 $$  h \triangleq sign(S_{k}),  $$

where *s*_*k*_ is the contribution value of *k*^*th*^ site that stands for contribution to the binding probability, *F*_*ijk*_ is the *j*^*th*^ hidden feature of *k*^*th*^ site (the *k*^*th*^ out of the whole length of peptide) in the *i*^*th*^ level. *W*_*ij*_ is the contribution weight for the *j*^*th*^ hidden feature of the *i*^*th*^ level. *m*_*i*_ stands for the *i*^*th*^ level contribution weights from the merge layer. Formula 3 shows how to get the precise site rank in each peptide. *h* is defined as the high contribution of each site. We define *h* in Formula 4 and we think the site which exhibits negative contribution to the affinity scores cannot be the motifs. Figure 3 shows the calculation process of low level’s sites contribution. Low-level CNN feature is output from the first convolutional layer, and weights are called from the well-trained network (the dotted line in purple).

#### Mutation

After getting the contribution of each site of the peptide, we can extract the motifs from the MHC-peptide binding mechanism. To generate a new peptide of high binding affinity, the next step is to preserve the motifs and mutate the other amino acids in the peptide. But before creating the new peptide, we need to explain a biological or chemical conclusion drawn by other scholars “The non-motifs sites contribute little to the MHC-Peptide binding [46]”.

According to this conclusion, if we fix these motifs and mutate other sites with other amino acids, we can generate new peptides with high binding affinity for sure. Specifically, we rank the score list of each site in a peptide and convert the amino acid on the site with the lowest score to other amino acids randomly. Notably, the peptides choosing for generation only are those predicted to be binders (that is, the value of IC50 is less than 500 nM).

### Transfer Learning

Through the method mentioned above, we can gain many new generational high binding affinity peptides to a specific MHC. However, how can we use this method in other MHC? Comparing with the known abundant peptide datasets of the HLA-A*0201, other MHC-peptide pairs are only discovered a little and the peptide amount is not sufficient that they cannot be trained from scratch (it will quickly cause overfitting). Thus, a new training method needs to be proposed for the MHC with a small dataset.

According to the property of MHC [46], some are related and similar (aka subtype) while others are alienated. This provides insights that we ought to use similar motifs to represent the binding mechanism of peptides and similar MHCs.

Basing that two structure similar MHC molecules have the similar binding mechanism to peptides, we can make a reasonable inference that two structure similar MHC molecules have similar motifs. Therefore, we decide to use two transfer learning method to the different MHC according to their relationship to the most significant dataset from allele HLA-A*0201. For the alienated MHC molecule, we take advantage of the fine-tune method while for the similar MHC, we exert the direct transfer method (also known as zero-shot learning method [47]).

## Results

We downloaded the MHC-I peptide datasets from the IEDB [48]. We filtered, processed, and prepared the data according to the guidelines in Vang’s work [10]. The amount of highly-binding-affinity peptides of different allele subtypes varies substantially. The 9-mer peptides of HLA-A*0201 are 13,088 while the 9-mer peptides for HLA-A*0206, HLA-B*2705, HLA-A*6802, and Mamu-A1*001:01 are only 3062, 1966, 3764 and 899, respectively. To demonstrate the MAM model, we choose the representative MHC alleles as HLA-A*0201, HLA-A*0206, HLA-B*2705, HLA-A*6802, and Mamu-A1*001:01 with 8-mer to 11-mer peptide lengths. These MHCs will help us to evaluate the model comprehensively.

We have two subproblems, the first is binding affinity predictions, and the second is peptide generation,

For MHC datasets in human such as HLA-A*0201, HLA-A*0206, HLA-B*2705, and HLA-A*6802, we use an IEDB independent dataset for both binding affinity prediction and peptide generation which leads to the proportion of training dataset and testing dataset is approximate to 0.99:0.01 (These details of IEDB set are introduced in [10]). The IEDB numbers of these datasets are IEDB 1029824, 1028790, IEDB 1029125 and IEDB 1028790 respectively.

For MHC datasets in animals (like Macaca rhesus), such as Mamu-A1*001:01, we split the dataset into 0.95:0.05 for binding affinity prediction and peptide generation; that is, 95% peptides belong to the training set and 5% of peptides belong to the testing set for prediction and generation. We slightly increase the proportion of testing dataset here in order to better evaluate the generation performance under these datasets. Basing the fact that inputting the training dataset for generating peptides will lead to over-performance, therefore testing dataset is inputted into the generator for convincing performance evaluation. As the datasets in Macaca rhesus (like Mamu-A1*001:01) are generally smaller than those in human, so we slightly increase the proportion of testing dataset from 0.01 to 0.05. As a consequence, we generate a reasonable amount of peptides for the following analysis and evaluation.

### Training details

The network is built with Keras library [49]. The program is run on a 1080ti. Most of the training process terminated within 400 epochs. Each model takes advantage of early stopping method with patience = 20, which means the training will stop when 20 epochs have no improvement. The training time is between 5 min to 10 min for 10 times random initialization. We also use *l*_2_ regularization (0.01) and dropout method to restrict kernels and avoid over-fitting. The details of our model are in Table 1.

**Table 1:** Architecture of MHC-CNN network

Type	Notes
Input layer	
Embedding(each site vec dim = 15)	Finally build N*15 matrix(N is the mer number in peptide, N is 9 denotes 9 mer)
Conv1D[filter_size=16, filter_length=7] + LeakyReLU(0.3)	Low-level feature
Dropout(0.25)	
Conv1D[filter_size=32, filter_length=7] + LeakyReLU(0.3)	High-level feature
Dense layer1(1) without bias	Global averaging Pooling network, input is the first Conv1D
Dense layer2(1) without bias	Global averaging Pooling network, input is the second Conv1D
Dense layer3(1) without bias	Fusion of the different level GAP layers(aka voting method)
Sigmoid [prediction]	

### Evaluation criteria

1. SRCC, AUC
  We use Spearman’s rank correlation coefficient (SRCC) and area under the receiver operating characteristic curve (AUC) to evaluate the performances.
2. high-affinity rate
  High-affinity rate depicts the proportion of high-affinity peptides among the total generated peptides, and the binding affinity values are from the result of IEDB prediction source
  (http://tools.iedb.org/mhci/). All the options are all default except the MHC allele type and peptide length. We regard IC50 is less than 500 nM as high-affinity peptides, which is the common conversion adopted by the community [10, 18]. We use the high-affinity rate as the evaluation criteria for generated peptides.

### Evaluation of network architectures

The evaluation of network architectures is shown in Tables 2 and 3. Table 3 shows, the highest AUC is from two convolutional layers with multiple feature fusion model. The SRCC score of 2CNN+FC is the highest among all the candidates, but the AUC is less than our proposed network (0.56 to 0.593).

**Table 2:** Binding Affinity Prediction Performances of different network architectures

Model	SRCC	AUC
2CNN+FC	0.178	0.56
2CNN + GAP	0.083	0.554
1CNN + GAP	0.119	0.575
2CNN + muti-GAP	0.117	0.576
3CNN + GAP	0.139	0.59

The training dataset is HLA-A*0201 while the test dataset is IEDB 1029824 HLA-A*0201 segmented from HLA-A*0201. FC denotes full-connected layer. SRCC stands for Spearman’s rank correlation coefficient and AUC stands for area under the receiver operating characteristic curve. All the models are well-trained. “A CNN+B GAP” represents A CNN layers and B Global Pooling Layers in the feature caught part. The “2CNN + muti-GAP” is our final MHC-CNN predictor

**Table 3:** Generation Performances of different network architectures

Model	high-affinity rate
2CNN + GAP	0.756
1CNN + GAP	0.813
2CNN + muti-GAP	0.859
3CNN + GAP	0.481
random data	0.05

The dataset is HLA-A*0201. Except for the random data model, all the model is a variety of MAM network. High-affinity rate denotes the fraction of peptides with high affinity in all the generated peptide. Random data is to create data randomly at all sites. GAP stands for global averaging pooling. “A CNN + B GAP” represents A numbers of CNN layer and B numbers of Global Pooling Layer in the feature extraction part. All the definitions we mentioned are the same

For the generation performance, comparing with random generation, all the models reach higher scores. **It provides us with insights that all the models we proposed have the ability to generate high-affinity peptides**.

As to the high-affinity rate, with the increment of the number of layers, the score decreases to a low value (from 0.813 to 0.481). The model fusion method can outperform others greatly. It is mainly because a fixed length CNN may extract feature with a certain size and it is inadequate to recognize the standard motifs which have a complex spatial relationship. Accordingly, we apply the multi-feature fusion method.

Moreover, the SRCC, AUC and the high-affinity rate are connecting tightly. High SRCC and AUC mean high-affinity which reveals that our model has ability to extract meaningful motifs.

### Evaluation of generated peptides between various k-mers and MHCs

We focus on the peptide generation problem in this work. As an intermediate result, we evaluate the binding affinity prediction. Table 4 demonstrated that our model outperforms the state of the art methods in terms of AUC. This indicated that the prediction model has a great potential in the generation or other relative areas. **The prediction and the generation problems own similar features.**

**Table 4:** Prediction performance comparison of our MHC-CNN with other networks

Model	SRCC	AUC
NetMHCpan [52]	0.071	0.546
sNebula [53]	0.06	0.539
HLA-CNN [10]	**0.178**	0.56
MHC-CNN	0.117	**0.576**

All the training dataset is HLA-A*0201 while the testing dataset is IEDB 1029824 HLA-A*0201 segmented from HLA-A*0201. MHC-CNN denotes our best performance network architecture: 2CNN+multi GAPs. The bold face denotes the best performance of the column

Table 5 displays the results of the zero-shot transfer learning with initial learning dataset as A*0201. The most significant improvement is the generated peptides for B*2705, and the second one is the peptides of Mamu-A1*00101. It is evident that the MHC has a farther distance to the A*0201 is the one which shared fewer motifs with A*0201. For examples, the results for A*0206 have a better performance than A*6802, the results of HLA-A alleles (A*0201, A0206, A*6802) are better than HLA-B (B*2705), and the human alleles are better than mammalian alleles (Mamu-A1*001:01). After fine-tuning, the transfer learning has a much better performance which suggests the models have a good extendibility; that is, and after fine-tuning, the farther MHC alleles from the initial dataset have a more significant improvement of the high-affinity rate and fine-tune method aids to catch the motifs to the specific MHC.

**Table 5:** Transfer learning methods for representative MHC alleles

MHC allel	SRCC	AUC	high affinity rate^1^	transfer learning method^2^	Improvement^3^
A*6802	0.499	0.817	0.9	fine-tune	0.61
	0.058	0.537	0.29	zero-shot	
A*0206	0.458	0.778	0.75	fine-tune	0.08
	0.378	0.73	0.67	zero-shot	
B*2705	0.701	0.929	0.92	fine-tune	0.92
	0.167	0.602	0	zero-shot	
Mamu-A1*001:01	0.755	0.943	0.839	fine-tune	0.809
	0.256	0.65	0.03	zero-shot	
A*0201	0.117	0.576	0.859	originally	

^*^The length of the peptides for training transfer learning is nine.

^1^The high-affinity is scored by http://tools.iedb.org/mhci/.

^2^There are two types of transfer learning. Fine-tune indicates keeping training on the basis of the original model with a smaller learning rate ($\frac {1}{10}$ learning rate). Zero-shot indicates that direct transferring without further training. Originally indicates the model is the well-trained model that all the transfer learning is based on.

^3^the Improvement stands for the increase of high-affinity rate from zero-shot transfer method to fine-tune method

**Fig. 4:**
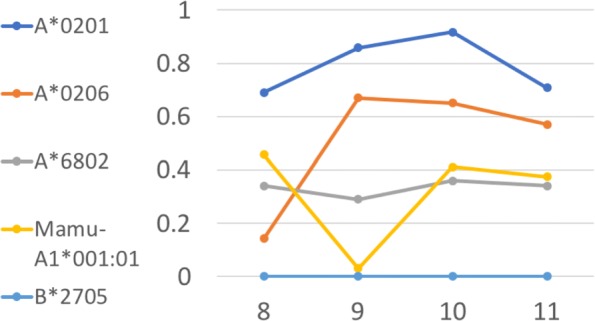
Performance of the generation method for different length peptides. X-axis represents the length of generated peptides while Y-axis is the high-affinity rate of generated peptides. Different colors indicate the peptides are generated from different MHC datasets. All the peptides are generated through zero-shot transfer learning upon the original trained model of A*0201 allele

Usually, the relations between human’s MHCs are tighter than those between human and animals. From Fig. 4, though HLA-A alleles still have higher high-affinity rates than B*2705 (HLA-B allele) and Mamu-A1*001:01 (rhesus macaque allele), Mamu-A1*001:01 have a higher high-affinity rate than HLA-B allele B*2705 with certain lengths. It mainly due to the HLA-B dataset with high-affinity is more insufficient than HLA-A dataset and datasets from mammiferous MHC alleles.

### Evaluation on the motifs extraction

**Fig. 5:**
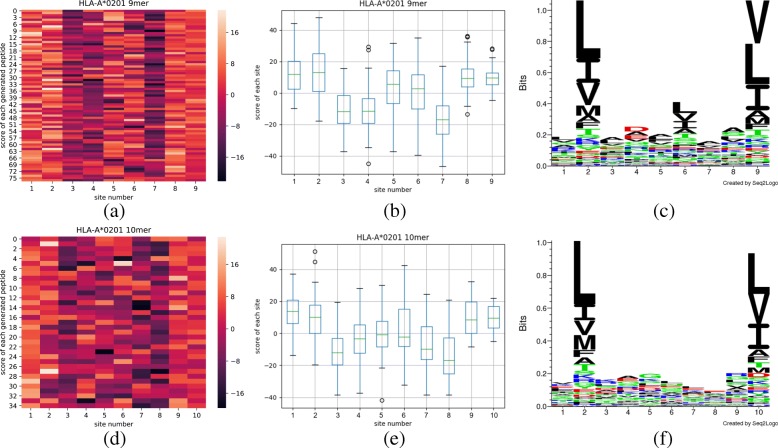
The top row represents heatmap (**a**), boxplot (**b**) and seq2logo (**c**) of HLA-A*0201 allele’s new peptides with 9 lengths generated from the well-trained network while the bottom row represents heatmap (**d**), boxplot (**e**) and seq2logo (**f**) of 10-mer peptides generated using fine-tune method corresponding to same allele. In heatmap, the horizontal axis indicates the site of peptide and the vertical axis indicate each peptide from the testing dataset, each pixel denotes the contribution of a certain site on the certain peptide to its binding affinity, where the lighter color is, the more contribution it represents. In the boxplot, each box collects all the contribution of peptides in the certain site and find out the average contribution to the binding affinity. We believe the site is important to this length peptide’s binding affinity if only the average is greater than zero. And in sequence logos, on the vertical, the logo distribution intuitively depicts the amino acid frequencies on each site of the peptides

Based on the results in Table 5, we collect the generated peptides to demonstrate the network ability in motifs extraction. As the training set is all from HLA-A*0201’s 9-mer dataset, we firstly generate 9-mer peptides, and after fine-tuning, we generate 10-mer peptides. To evaluate the performance of motifs extraction, we use all the generated peptides to produce the heatmap, boxplot, and sequence logos as shown in Fig. 5.

As shown in Fig. 5a, we can observe the color is much lighter in columns 1, 2, 8 and 9 than the color in column 3, 4 and 7, which suggests in the most generated peptides with 9 lengths, the site 1, 2, 8 and 9 contributed more important to the binding affinity, and the sites 3, 4 and 7 contributed less to the binding affinity.

Compared with Fig. 5b, we can find that sites 1, 2, 8 and 9, which are regarded as more important from heatmap. The site 3, 4 and 7 contributed less, the average is less than zero. The boxplot also support the observations. After analyzing from heatmap and boxplot, we can quickly conclude how each site influences on the binding affinities of the peptides to HLA-A*0201.

In sequence logo of Fig. 5c, we can conclude the amino acid frequency of each site, which suggests the amino acid contributions in each site to the binding affinity, which is directly called from network’s training. Combining Fig. 5a, b, c, we can figure out the specific amino acids at certain sites of 9-mer peptide contributed to the binding affinity, which we called the motifs. For example, to site 9, Valine (“V”) is the most contributory while Leucine (“L”) and Isoleucine (“I”) rank the second and third, respectively. We can conclude Leucine (“L”) at site 2, Valine (“V”) and Leucine (“L”) at site 9 largely influence the binding affinity between 9-mer peptides and HLA-A*0201.

Similarly analyzing on the 10-mer peptides from HLA-A*0201, combining Fig. 5d, e, f we can figure out the important site as 1, 2, 9 and 10. At site 2, Leucine (“L”) is much important, while to site 10, Leucine (“L”) and Valine (“V”) are both important.

**Fig. 6:**
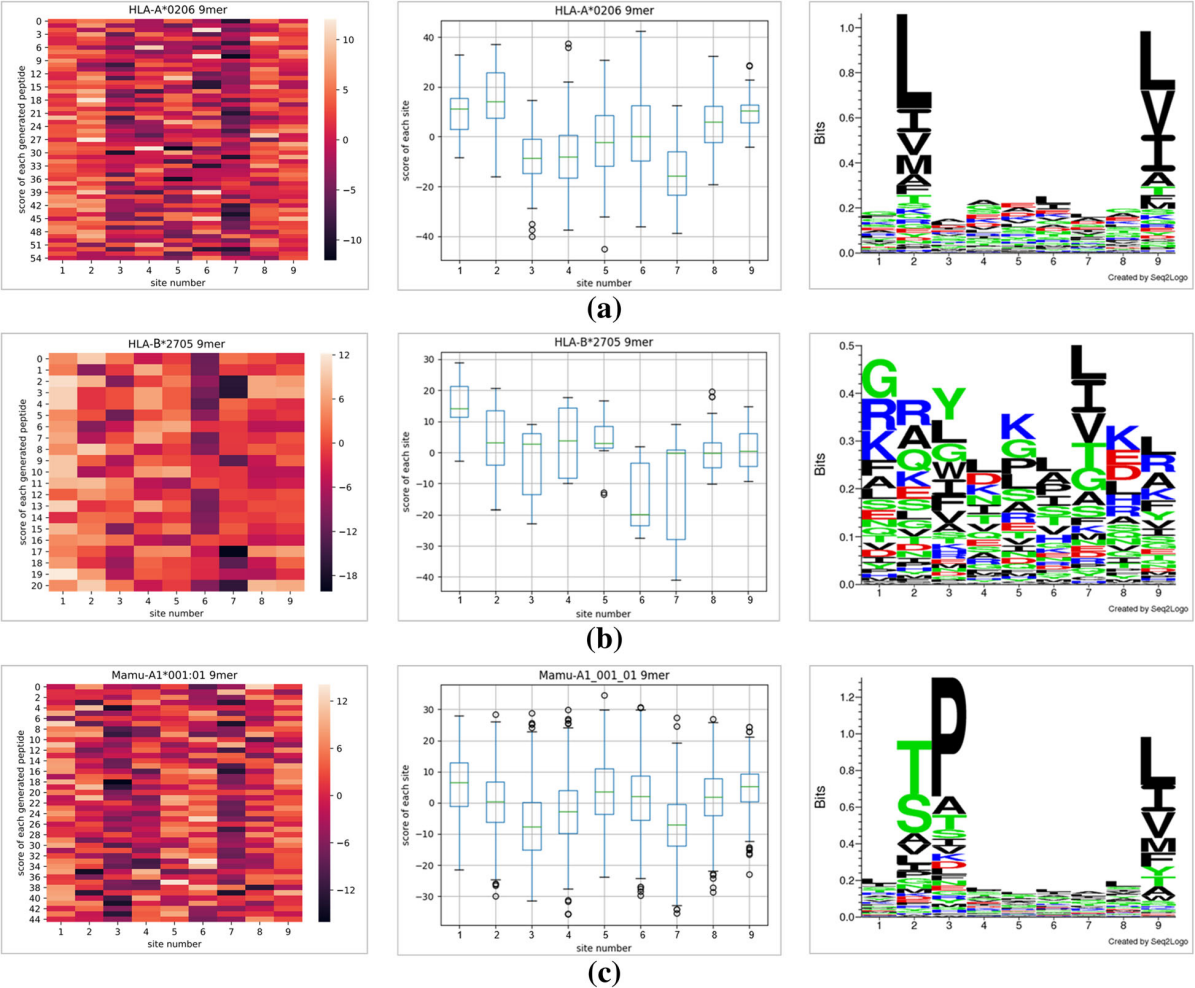
The heatmaps, boxplots and seq2logoes of HLA-A*0201, HLA-A*0206, HLA-B*2705 and Mamu-A1*001:01 allele’s new 9-mer peptides with 9 lengths generated from the well-trained network using fine-tune method. After separately fine-tuning from the well-trained network, we generate some 9-mer peptides with high-affinity to certain representative MHC, and they are (**a**) HLA-A*0206, (**b**) HLA-B*2705 and (**c**) Mamu-A1*001:01. The instructions of heatmap, boxplot and sequence logos see Fig. 5’s legend in detail

Figure 5 shows motifs extraction by the network for the HLA-A*0201 dataset. To understand the motifs of other MHC dataset, we collect the HLA-A*0206 9-mer, HLA-B*2705 9-mer, and Mamu-A1*001:01 9-mer datasets to separately fine tune the present network and generate the peptides. We also use these peptides to produce the heatmap, boxplot and sequence logos as shown in Fig. 6.

From Fig. 6a, Leucine (“L”) at site 2, Leucine (“L”) and Valine (“V”) at site 9 largely contribute to the binding affinity between 9-mer peptides and HLA-A*0206. From Fig. 6b, the number of sites with average positive score is about 5, and the sequence logo shows great variety at each site (every amino acid’s frequency distribute evenly), so the motifs of HLA-B*2705 are numerous and unlike motifs of HLA-A*0201 and HLA-A*0206. Figure 6c indicates only Leucine (“L”) and Isoleucine (“I”) at site 9 largely influence the binding affinity between 9-mer peptides and Mamu-A1*001:01. The Threonine (“T”) at site 2 and Proline (“P”) at site 3 are outstanding in sequence logos, however, they do not contribute to the high-affinity, as we can conclude from the heatmap and boxplot that the site 2 does not have positive contribution to the binding affinity.

Separately compared with the observed motifs from HLA-A*0201 9-mer peptides, the motifs in HLA-A*0201 (Leucine at site 2, Valine and Leucine at site 9) and HLA-A*0206 (Leucine at site 2, Leucine and Valine at site 9) are very close. But motifs of HLA-B*2705 and Mamu-A1*001:01 much differ from motifs of HLA-A*0201. Basing HLA-A*0201 and HLA-A*0206 both belong to same supertype HLA-A2 because of they share common binding features to peptides, we think the motifs extracted from our network are similar to the features and they are in accordance with the aggregation of supertype A2.

## Discussion

In this section, we would like to discuss the effectiveness of deep learning in MHC-Peptide binding issue.

### Is deep learning suitable for an MHC-Peptide binding problem?

We think this answer is yes. But we ought to use them in a more reasonable and circumspect way rather than abuse this method. As far as we know, deep learning methods do not outperform the traditional method greatly [41] and if those who do not be familiar with the parameter tuning, he may probably get a worse result. Moreover, due to the limitation of the data, deep learning method are consed.

But why we still focus on the deep learning method? The answer is the explainability of deep learning. With the help of feature visualization methods, we can visualize the relation between various locations which can not be easily drawn from a human. That is one of the advantage of deep learning.

## Conclusion

### Summary

We design the network for both predicting the binding probability and extracting motifs to produce new peptides. Also, our experiment demonstrates that our algorithm can generate new peptides with high binding affinity, which in turn indicates motifs are available and reasonable with good performance.

### Future work

As for the future work, the proposed topics are as follows:

- Expanding the application of the network to peptides of MHC-II, basing the core combination region in the binding between MHC-II and peptides, I’m sure the performance will be perfect in motifs extraction.
- Improving the generation method. The present generated approach largely depends on the present peptides data, what if directly generating new peptides after learning the binding motifs? We think using more advanced generators to help with peptides generation will be the next objective for further researchers. For example, generative adversarial network [50] and adversarial autoencoder [51].
- Adding more information of the binding between MHC and peptides for better modeling the MHC-peptide binding mechanism, e.g. MHC sequence and PDB structure’s information [21].

## References

[1] Pandey JP (2007). Major histocompatibility complex. Med Immunol.

[2] Corr M, Slanetz AE, Boyd LF, Jelonek MT, Khilko S, Al-Ramadi BK, Kim YS, Maher SE, Bothwell A, Margulies DH (1994). T cell receptor-mhc class i peptide interactions: affinity, kinetics, and specificity. Science.

[3] Ekins S (2016). The next era: Deep learning in pharmaceutical research. Pharm Res.

[4] Skwark MJ, Raimondi D, Michel M, Elofsson A (2014). Improved contact predictions using the recognition of protein like contact patterns. PLoS Comput Biol.

[5] Zheng J, Zhang X, Zhao X, Tong X, Hong X, Xie J, Liu S (2018). Deep-rbppred: Predicting rna binding proteins in the proteome scale based on deep learning. Sci Rep.

[6] Quang D, Xie X (2016). Danq: a hybrid convolutional and recurrent deep neural network for quantifying the function of dna sequences. Nucleic Acids Res.

[7] Kelley DR, Snoek J, Rinn JL (2016). Basset: learning the regulatory code of the accessible genome with deep convolutional neural networks. Genome Res.

[8] Zhou J, Troyanskaya OG (2015). Predicting effects of noncoding variants with deep learning–based sequence model. Nat Methods.

[9] Lanchantin J, Singh R, Lin Z, Qi Y. Deep motif: Visualizing genomic sequence classifications. arXiv preprint arXiv:1605.01133. 2016.

[10] Vang YS, Xie X (2017). Hla class i binding prediction via convolutional neural networks. Bioinformatics.

[11] Bhattacharya R, Tokheim C, Sivakumar A, Guthrie VB, Anagnostou V, Velculescu VE, Karchin R. Prediction of peptide binding to mhc class i proteins in the age of deep learning. bioRxiv. 2017. 10.1101/154757. http://arxiv.org/abs/https://www.biorxiv.org/content/early/2017/06/23/154757.full.pdf.

[12] O’Donnell T, Rubinsteyn A, Bonsack M, Riemer A, Hammerbacher J (2018). Mhcflurry: open-source class i mhc binding affinity prediction. Cell Syst.

[13] Jurtz V, Paul S, Andreatta M, Marcatili P, Peters B, Nielsen M (2017). Netmhcpan-4.0: Improved peptide–mhc class i interaction predictions integrating eluted ligand and peptide binding affinity data. J Immunol.

[14] Nielsen M, Lundegaard C, Blicher T, Lamberth K, Harndahl M, Justesen S, Røder G, Peters B, Sette A, Lund O (2007). Netmhcpan, a method for quantitative predictions of peptide binding to any hla-a and-b locus protein of known sequence. PloS ONE.

[15] Reche PA, Glutting J-P, Reinherz EL (2002). Prediction of mhc class i binding peptides using profile motifs. Hum Immunol.

[16] Nielsen M, Lundegaard C, Worning P, Lauemøller SL, Lamberth K, Buus S, Brunak S, Lund O (2003). Reliable prediction of t-cell epitopes using neural networks with novel sequence representations. Protein Sci.

[17] Peters B, Sette A (2005). Generating quantitative models describing the sequence specificity of biological processes with the stabilized matrix method. BMC Bioinformatics.

[18] Karosiene E, Rasmussen M, Blicher T, Lund O, Buus S, Nielsen M (2013). Netmhciipan-3. 0, a common pan-specific mhc class ii prediction method including all three human mhc class ii isotypes, hla-dr, hla-dp and hla-dq. Immunogenetics.

[19] Mazzaferro C. Predicting protein binding affinity with word embeddings and recurrent neural networks. bioRxiv. 2017;:128223. https://www.biorxiv.org/content/early/2017/04/18/128223.abstract.

[20] Han Y, Kim D (2017). Deep convolutional neural networks for pan-specific peptide-mhc class i binding prediction. BMC Bioinformatics.

[21] Ragoza M, Hochuli J, Idrobo E, Sunseri J, Koes DR (2017). Protein–ligand scoring with convolutional neural networks. J Chem Inf Model.

[22] Gawehn E, Hiss JA, Schneider G (2016). Deep learning in drug discovery. Mol Inform.

[23] Kadurin A, Aliper A, Kazennov A, Mamoshina P, Vanhaelen Q, Khrabrov K, Zhavoronkov A (2017). The cornucopia of meaningful leads: Applying deep adversarial autoencoders for new molecule development in oncology. Oncotarget.

[24] Kadurin A, Nikolenko S, Khrabrov K, Aliper A, Zhavoronkov A (2017). drugan: an advanced generative adversarial autoencoder model for de novo generation of new molecules with desired molecular properties in silico. Mol Pharm.

[25] Segler MH, Kogej T, Tyrchan C, Waller MP (2017). Generating focused molecule libraries for drug discovery with recurrent neural networks. ACS Central Sci.

[26] Hautier G, Fischer C, Ehrlacher V, Jain A, Ceder G (2010). Data mined ionic substitutions for the discovery of new compounds. Inorg Chem.

[27] Schwaller P, Gaudin T, Lanyi D, Bekas C, Laino T. found in translation: Predicting outcome of complex organic chemistry reactions using neural sequence-to-sequence models. arXiv preprint arXiv:1711.04810. 2017. https://arxiv.org/abs/1711.04810. 10.1039/c8sc02339e PMC6053976 30090297

[28] Liu B, Ramsundar B, Kawthekar P, Shi J, Gomes J, Luu Nguyen Q, Ho S, Sloane J, Wender P, Pande V (2017). Retrosynthetic reaction prediction using neural sequence-to-sequence models. ACS Central Sci.

[29] Nielsen M, Andreatta M (2017). Nnalign: a platform to construct and evaluate artificial neural network models of receptor–ligand interactions. Nucleic Acids Res.

[30] Shrikumar A, Greenside P, Kundaje A. Learning important features through propagating activation differences. arXiv preprint arXiv:1704.02685. 2017. https://arxiv.org/abs/1704.02685.

[31] Alvarez B, Barra C, Nielsen M, Andreatta M. Computational tools for the identification and interpretation of sequence motifs in immunopeptidomes. Proteomics. 2018; 18(12):1700252. 10.1002/pmic.201700252. 10.1002/pmic.201700252 PMC6279437 29327813

[32] Mikolov T, Chen K, Corrado G, Dean J. Efficient estimation of word representations in vector space. arXiv preprint arXiv:1301.3781. 2013. https://arxiv.org/abs/1301.3781.

[33] Mikolov T, Sutskever I, Chen K, Corrado GS, Dean J (2013). Distributed representations of words and phrases and their compositionality. Proceedings of the 26th International Conference on Neural Information Processing Systems - Volume 2. NIPS’13.

[34] Le Q, Mikolov T. Distributed representations of sentences and documents. In: International Conference on Machine Learning. JMLR: 2014. p. 1188–96.

[35] Neelakantan A, Shankar J, Passos A, McCallum A. Efficient non-parametric estimation of multiple embeddings per word in vector space. arXiv preprint arXiv:1504.06654. 2015. https://arxiv.org/abs/1504.06654.

[36] Asgari E, Mofrad MR (2015). Continuous distributed representation of biological sequences for deep proteomics and genomics. PloS ONE.

[37] Falk K, Rötzschke O, Stevanovié S, Jung G, Rammensee H-G (1991). Allele-specific motifs revealed by sequencing of self-peptides eluted from mhc molecules. Nature.

[38] Falk K, Rötzschke O (1993). Consensus motifs and peptide ligands of mhc class i molecules. Seminars in Immunology, Vol. 5.

[39] Rötzschke O, Falk K (1991). Naturally-occurring peptide antigens derived from the mhc class-i-restricted processing pathway. Immunol Today.

[40] Bach S, Binder A, Montavon G, Klauschen F, Müller K-R, Samek W (2015). On pixel-wise explanations for non-linear classifier decisions by layer-wise relevance propagation. PloS ONE.

[41] Bhattacharya R, Sivakumar A, Tokheim C, Guthrie VB, Anagnostou V, Velculescu VE, Karchin R. Evaluation of machine learning methods to predict peptide binding to mhc class i proteins. bioRxiv. 2017;:154757. https://www.biorxiv.org/content/early/2017/07/27/154757.abstract.

[42] Yu S, Jia S, Xu C (2017). Convolutional neural networks for hyperspectral image classification. Neurocomputing.

[43] Witten IH, Frank E, Hall MA, Pal CJ (2016). Data Mining: Practical Machine Learning Tools and Techniques.

[44] De Boer P-T, Kroese DP, Mannor S, Rubinstein RY (2005). A tutorial on the cross-entropy method. Ann Oper Res.

[45] Zhou B, Khosla A, Lapedriza A, Oliva A, Torralba A (2016). Learning deep features for discriminative localization. Computer Vision and Pattern Recognition (CVPR), 2016 IEEE Conference On.

[46] Rammensee H-G, Bachmann J, Stevanovic S (2013). MHC Ligands and Peptide Motifs.

[47] Lampert CH, Nickisch H, Harmeling S (2014). Attribute-based classification for zero-shot visual object categorization. IEEE Trans Pattern Anal Mach Intell.

[48] Kim Y, Ponomarenko J, Zhu Z, Tamang D, Wang P, Greenbaum J, Lundegaard C, Sette A, Lund O, Bourne PE (2012). Immune epitope database analysis resource. Nucleic Acids Res.

[49] Chollet F, et al.Keras. GitHub. 2015.

[50] Goodfellow I, Pouget-Abadie J, Mirza M, Xu B, Warde-Farley D, Ozair S, Courville A, Bengio Y, Ghahramani Z, Welling M, Cortes C, Lawrence ND, Weinberger KQ (2014). Generative adversarial nets. Advances in Neural Information Processing Systems 27.

[51] Makhzani A, Shlens J, Jaitly N, Goodfellow I, Frey B. Adversarial autoencoders. arXiv preprint arXiv:1511.05644. 2015. https://arxiv.org/abs/1511.05644.

[52] Hoof I, Peters B, Sidney J, Pedersen LE, Sette A, Lund O, Buus S, Nielsen M (2009). Netmhcpan, a method for mhc class i binding prediction beyond humans. Immunogenetics.

[53] Luo H, Ye H, Ng HW, Sakkiah S, Mendrick DL, Hong H (2016). snebula, a network-based algorithm to predict binding between human leukocyte antigens and peptides. Sci Rep.

